# The Use of Wooden Clubs and Throwing Sticks among Recent Foragers: Cross-Cultural Survey and Implications for Research on Prehistoric Weaponry

**DOI:** 10.1007/s12110-023-09445-3

**Published:** 2023-03-29

**Authors:** Václav Hrnčíř

**Affiliations:** 1 Department of Linguistic and Cultural Evolution, Max Planck Institute for Evolutionary Anthropology, Leipzig, Germany; 2 Institute of Archaeology, Czech Academy of Sciences, Prague, Czech Republic

**Keywords:** Comparative ethnology, Hunter-gatherers, Pleistocene archaeology, Weapons, Wooden clubs, Throwing sticks

## Abstract

There is a popular idea that archaic humans commonly used wooden clubs as their weapons. This is not based on archaeological finds, which are minimal from the Pleistocene, but rather on a few ethnographic analogies and the association of these weapons with simple technology. This article presents the first quantitative cross-cultural analysis of the use of wooden clubs and throwing sticks for hunting and violence among foragers. Using a sample of 57 recent hunting-gathering societies from the Standard Cross-Cultural Sample, it is shown that the majority used clubs for violence (86%) and/or hunting (74%). Whereas in hunting and fishing the club usually served only as a secondary tool, 33% of societies used the club as one of their main fighting weapons. The use of throwing sticks was less frequent among the societies surveyed (12% for violence, 14% for hunting). Based on these results and other evidence, it is argued that the use of clubs by early humans was highly probable, at least in the simplest form of a crude stick. The great variation in the forms and use of clubs and throwing sticks among recent hunter-gatherers, however, indicates that they are not standardized weapons and that similar variation may have existed in the past. Many such prehistoric weapons may therefore have been quite sophisticated, multifunctional, and carried strong symbolic meaning.

**Supplementary Information:**

The online version contains supplementary material available at 10.1007/s12110-023-09445-3.

Archaic humans, such as early *Homo* *sapiens* or *Homo* *neanderthalensis*, are often portrayed in popular culture as stereotypical cavemen dwelling in caves, wearing animal skins, and wielding large wooden clubs.^1^ The aim of this paper is to discuss the latter attribute. Could the wooden club really have been a common Pleistocene weapon or is it a myth? What practical use could it have had? And how sophisticated a form and meaning could it take?

In order to answer these questions, I conducted quantitative cross-cultural analysis of the use of wooden clubs for hunting and violence among recent foragers. Although clubs can be made of different materials (e.g., wood, bone, antler, stone, metal) and be composed of several parts (e.g., a mace with the head attached to a shaft), this article focuses primarily on the simplest type—the one-piece all-wooden club. I expanded my research to include throwing sticks—in other words, projectiles made of one or several wood pieces that are launched by hand to hit a target in the manner of a blunt weapon (Bordes, 2014, 2020). In theory, these weapons are different in shape and function. However, in reality, some clubs were also used as projectiles, whereas some throwing sticks may serve in part as contact weapons (Bordes, 2014; Davidson, 1936). Moreover, both weapons share some overlapping characteristics, and sometimes it is hard to discriminate between them and to say definitely where the club ends and the throwing stick begins (Basedow, 1913:300).

The cross-cultural survey is supplemented by a review of the use of sticks and clubs by modern nonhuman primates. Finally, I discuss the practical use of clubs and throwing sticks, especially in comparison with other weapon systems.

## Archaeological Evidence of Wooden Clubs and Throwing Sticks

The first argument against the existence of clubs in the Pleistocene is their near absence in the archaeological record (e.g., Gamble, 2001:6; Stoczkowski, 2002:79). Unlike wooden spears and lances (e.g., Oakley et al., 1977; Schoch et al., 2015; Thieme & Veil, 1985), only one club-like weapon from the Paleolithic period has been found. A short, heavy piece of wood dated to the Acheulean period and interpreted as a throwing club or mallet was discovered at the site of Kalambo Falls, Zambia (Clark, 2001:484). Mesolithic findings are also rare. Exceptions are two wooden clubs from Holmegaard Moor, Denmark, dated to around 6,500–6,000 BC (Brøndsted, 1960:72). More evidence comes from later periods, especially Neolithic and Bronze Age moorland, lakeshore, and riverside settlements. In total, about 40 wooden clubs have been discovered across 19 sites in Denmark, Germany, the Netherlands, Switzerland, and England (Strambowski, 2015). Later use of clubs is proved by findings from late Bronze Age and Early Iron Age lake settlements in Poland, as well as from German and Danish sites dated to the first millennium BC (Kontny, 2015). Non-European finds include the throwing club or knobkerrie from Later Stone Age Namibia (Clark & Walton, 1962; Wadley, 2012); clubs, throwing sticks, and boomerangs from ancient Egypt (Reeves, 2007:175–76; Pitt-Rivers, 1883); and two wooden fish clubs from prehistoric Oregon (Minor & Nelson, 2004).

Finds of throwing sticks and boomerangs are older, but Pleistocene finds are also rare. Two 300,000-year-old double-pointed wooden sticks possibly used as throwing sticks were discovered in Schöningen, Germany (Conard et al., 2020; Thieme, 1997). Another, albeit non-wooden, specimen made of a mammoth tusk comes from an Upper Pleistocene site (23,000 BP) in south Poland (Valde-Nowak et al., 1987). The oldest Australian boomerangs known from Wyrie Swamp are dated to the early Holocene, 10,200–8,900 BP (Luebbers, 1975), although Australia’s boomerang stencil rock art is several thousand years older (Finch et al., 2021). Similarly old (9,000 BP) is a portion of a nonreturning oak boomerang found at Little Salt Spring, Florida (Clausen et al., 1979). (For an overview of finds from later periods, see Bordes, 2014:6–13.)

Although ethnographic literature shows that wooden clubs were commonly used in warfare (see below), only two specimens have been recovered in the direct context of a military conflict, coming from a Bronze Age battlefield in the Tollense Valley, Germany (Jantzen et al., 2011). Nevertheless, the prehistoric use of clubs for interpersonal violence is suggested indirectly by common evidence of blunt force cranial trauma. Wooden clubs are among the suspected weapons, for example, in the case of the death of an Upper Paleolithic man from Cioclovina cave, Romania (Kranioti et al., 2019); victims in Neolithic mass graves in Asparn/Schletz, Austria, and Talheim, Germany (Teschler-Nicola et al., 1999:443–44; Wahl & Trautmann, 2012:84–85); victims in a Copper Age mass grave from Potočani, Croatia (Janković et al., 2017:138–39); several individuals from sub-Neolithic cemeteries in Gotland, Sweden (Ahlström & Molnar, 2012:28); and Neolithic to Iron Age sites in Britain (Schulting & Bradley, 2013:49; Schulting & Wysocki, 2005:125; Smith, 2014:112). The link between some of these injuries and wooden clubs has been supported experimentally (Dyer & Fibiger, 2017). Beyond Europe, evidence for wounds consistent with club strikes comes also from late Pleistocene Kenya (Mirazón Lahr, 2016; Mirazón Lahr et al., 2016), prehistoric California and Colorado (Lambert, 2014; Walker, 1989), and precolumbian Chile (Lessa & Mendonça de Souza, 2004).

Iconographic depictions, such as an Early Bronze Age rock art killing scene in Medbo, Sweden (Toreld, 2012: Fig. 1); a hunting scene on Iron Age situla from Welzelach, Austria (Lucke & Frey, 1962: Fig. 76); and Roman stelae, gravestones, and columns (Kontny, 2015; Speidel, 1993) provide another type of evidence. In these cases, however, it is often difficult to prove that the weapons depicted are indeed wooden clubs (and not maces with stone or metal heads). The characteristic curved shape of many throwing sticks offers more certainty in this respect (for a review of their rock art representations, see Bordes, 2014:20–28).

Why is evidence for all-wooden clubs and throwing sticks from the Pleistocene so rare? There are several reasons, including poor preservation of organic materials, difficulty of determining function, and the often uncharacteristic shape of such weapons. On the one hand, any unworked piece of wood could serve as a club; on the other, any club-like artifact may have been used for hunting and violence as well as for cracking nuts. An example is the wooden fragment from Florisbad, South Africa, at least 125,000 years old. Although originally interpreted as the grip end of the throwing club, a subsequent reassessment concluded that a determination of its function is impossible (Bamford & Henderson, 2003). Similarly, the function of one of the Schöningen “throwing sticks” is also inconclusive (Schoch et al., 2015).

However, even if we accept that the absence of evidence is not evidence of absence, there is a second argument against the regular use of clubs in the Pleistocene, made for example in the podcast *Every* *Little* *Thing*, episode “Caveman Confidential.”^2^ It points to the fact that early humans were far more sophisticated than to rely on such a “simple” tool as the club. It has been argued that the club had limited usefulness in hunting game and could serve as a primary weapon only in the form of a throwing stick, especially a boomerang (Forde, 1955:155–56).

Before we verify or refute this claim, let us first examine where the idea of the club as the basic weapon of prehistoric people came from.

## The Club as a Weapon of Prehistoric People: The History of the Idea

In the nineteenth century, a series of archaeological discoveries provided the first evidence that humankind is older than the biblical six thousand years (Grayson, 1983). As a result, the first scientific ideas about the life of prehistoric humans began to emerge. Perhaps surprisingly, clubs were not always part of these reconstructions. On the contrary, they were depicted only in a minority of cases (see Table S1 in the Electronic Supplementary Material [ESM]).^3^ In the earliest depictions, prehistoric humans, although pre-sapient in appearance, were equipped with a stone axe (e.g., Boitard, 1838:209, 1861: cover; Huxley’s sketch from 1864). The club in the hands of prehistoric human probably first appeared in 1870 in the figure *Man* *in* *the* *Great* *Bear* *and* *Mammoth* *Epoch* (Figuier, 1870: Fig. 16). Nevertheless, some hints of a club (or at least a stick) can be found earlier in two Edenic scenes depicting the first humans, Adam and Eve (Figuier, 1863: Fig. 310; Unger, 1847: pl. XIV). The pair of images of the Neanderthal from La Chapelle-aux-Saints illustrate well the variability of the first reconstructions, including the form of the earliest weapons (Moser, 1992; Sommer, 2006). Whereas František Kupka depicted him with a simple weapon, probably a wooden or bone club (Reichart, 1909: Fig. 1), Amédée Forestier’s Neanderthal is armed with composite tools—a stone axe and a stone-tipped spear (Keith, 1911: Fig. 2). The most iconic depictions of Neanderthals with a club include an illustration in the 1915 book *Men* *of* *the* *Old* *Stone* *Age* (Osborn, 1915: pl. I) and one of the dioramas in the Field Museum of Natural History in Chicago (Field, 1933: pl. II).

In general, although never found, the club was perceived as a simple weapon that was logically suited to the hands of prehistoric human. This idea can be traced back to the texts of Greco-Roman authors of the first century BC. For example, Roman philosopher Lucretius in his *De* *Rerum* *Natura* writes that “The human beings who lived on earth in those early days... pursued the wild beasts of the forests with sling-stones and ponderous clubs” (Lucretius, *On* *the* *Nature* *of* *Things*, 5.925–5.969; see also Horace, *Satires*, 1.3.101; Diodorus Sicilus, *Library* *of* *History,* 1.24.3). Of course, these authors did not describe human prehistory as we understand it today; instead they populated the distant past with mythological figures and creatures (Moser, 1998:21–38). Along with giants, centaurs, and satyrs, the most prominent character armed with a club was the Herakles/Hercules, an archetype of a mythical warrior frequently depicted in combat with monsters and wild animals. Ancient authors also described clubs as common weapons of barbarian tribes (Ammianus Marcellinus, *Roman* *History*, 31.7.12; Diodorus Sicilus, *Library* *of* *History,* 3.8.24; Herodotus, *Histories* 7.63.1, 7.69.1; Tacitus, *Germania*, 45). However, the extent to which this was an accurate description of reality rather than a stereotypical image of barbarians is uncertain (Kontny, 2015).

Several centuries later, the club became the typical weapon of the “wild man,” first appearing in the thirteenth century AD (Moser, 1998:48–52). Although his image was clearly influenced by classical sources, the wild man was not seen as a primal ancestor but belonged to the realm of European folklore. The club, together with hairiness and nakedness, signified his wildness and the antithesis of civilization. The wild man and wild woman were very popular figures, appearing not only in medieval literature and art, but also in various carnivals (Bernheimer, 1952), and they undoubtedly contributed to later ideas about prehistoric “cavemen” (Stoczkowski, 2002:79–82).

Whether the influence on paleoanthropological reconstructions has been direct, however, is not clear. By the end of the seventeenth century, wild people all but disappeared from popular discourse, and the club began to appear more in the hands of nonhuman primates and “exotic” non-Western peoples (Forth, 2007; Laskow, 2015). While the former were often depicted holding staves or clubs to help them walk upright (see Yerkes & Yerkes, 1929:8–26), ethnographic analogies provided evidence of the actual use of clubs as ceremonial or real weapons. Comparison of several Indigenous societies subsequently enabled Lubbock to state that the club and the spear “seem to be the only natural and universal weapons of man” (Lubbock, 1865:475; also Darwin, 1871:234).

In the following sections, I will explore this “universality” in more detail using quantitative cross-cultural methods (Ember & Ember, 2009), instead of mere analogies, and compare the club with other weapon systems.

## The Use of Clubs and Throwing Sticks among Recent Hunter-Gatherers

### Material and Methods

The main data source for this study was *eHRAF* *World* *Cultures,* an online full-text database containing ethnographic collections of more than 350 cultures developed by the Human Relations Area Files (HRAF).^4^ eHRAF is unique among other databases in having subject indexing at the paragraph level based on the *Outline* *of* *Cultural* *Materials* (OCM) thesaurus (Murdock et al., 2008), which allows for detailed and more precise searching for concepts not easily found with keywords.

The *Standard* *Cross-Cultural* *Sample* (SCCS), which was developed to give equal weight to each culture area in the world (Murdock & White, 1969), was chosen as the sampling frame. All the SCCS cases are now included in eHRAF. Out of the 186 SCCS societies, only those that the HRAF staff coded as *hunter-gatherers* (defined as societies that “depend almost entirely [86% or more] on hunting, fishing, and gathering for subsistence”) or *primarily* *hunter-gatherers* (depending “mostly [56% or more] on hunting, fishing, and gathering for subsistence”) were included in the study. As a result, the final sample consists of 57 foraging societies, of which 39 are *hunter-gatherers* and 18 are *predominantly* *hunter-gatherers*.

For the purposes of this study, a *club* is defined as an all-wooden blunt weapon used primarily for striking at short distances. In its simplest form it is an unworked stick; in its most advanced form it is a specially shaped and decorated bludgeon. A *throwing* *stick* is defined as an all-wooden weapon that is primarily used as a projectile. It is thrown by hand and hits a target as a blunt weapon (which distinguishes it from a throwing spear that pierces a target). Any unworked stick can be used as a single-use throwing stick. However, specialized throwing sticks (including boomerangs) are characterized by a whole set of aerodynamic characteristics and come in a variety of shapes (Bordes, 2014). Unfortunately, *throwing* *sticks* were often only briefly mentioned without any detailed description in the analyzed literature. Therefore, in many cases it was unclear whether a specialized weapon was used for throwing or whether it was the same *club/stick* used for beating. Given these difficulties, my analyses focus primarily on the method of use (clubbing vs. throwing) and not on the type of weapon (club vs. throwing stick).

Since the *club* and the *throwing* *stick* can be described by various words (stick, cudgel, bludgeon, mace, bat, knobkerrie, rungu, boomerang, waddy, etc.), to identify its presence and use among selected societies, it was necessary to use OCM identifiers instead of simple keyword searching. Several OCM identifiers associated with weapons and hunting practices were used, specifically *Fowling* (223), *Hunting* *and* *trapping* (224), *Marine* *hunting* (225), *Fishing* (226), *Fishing* *gear* (227), and *Weapons* (411). This was supplemented by a keyword search for the words *club,* *stick,* *cudgel*, *bludgeon* and their variants (clubs, sticks, clubbing, bludgeoned, etc.) using terms “club*”, “stick*”, “cudgel*” and “bludgeon*”. The Boolean OR operator was used between OCMs, as well as between subject and keyword search.

For each society, I read all of the text results and extracted the relevant information into the dataset available on the Open Science Framework at https://osf.io/wydsj (hereafter referred to as Dataset). I recorded the use of clubs/sticks for five activities: *land* *animal* *hunting,* *bird* *hunting,* *marine* *mammal* *hunting,* *fishing,* and *interpersonal* *violence* *or* *warfare*. For each of these activities, I further recorded whether they were used for *clubbing* (i.e., as contact weapons) or *throwing* (i.e., as projectile weapons), and whether they were used as primary or secondary weapons. *Primary* means that the club/stick was used regularly for the activity as the main weapon, or one of the main weapons. *Secondary* means that it was not the main weapon and was used only occasionally for the activity. Sometimes it was not clear from the literature whether the weapon was used regularly or only occasionally; in this case the code *Primary?* was used. In case of ambiguous or conflicting information, the activity was coded with a question mark. In the case of hunting and fishing activities, the method of use was also recorded, namely whether the club/stick was used for hunting (i.e., catching and killing) or only for finishing off prey caught by other means (e.g., shot with a bow or spear, caught in a trap), and which specific animals were hunted with it (Appendix). If the verb “to club” was mentioned but it was not clear whether a *club* (as defined above) was actually used in this context, then this information was considered insufficient and was not recorded. If the search results provided no mention of clubs or throwing sticks, I read all the sections on hunting, fishing, warfare, and weapons in the major ethnographies on that culture (see references in the Dataset), and if I found no mention there either, *no* *evidence* was inferred for that case. *Digging* *sticks* were not considered clubs (although they could be used in the same way) because their primary function is different. However, if a digging stick (or other instrument such as a paddle) was used as a weapon, this fact was also recorded. I have also recorded when the club, or throwing stick, was used as a multifunctional weapon—simultaneously for clubbing/throwing, or for violence/hunting. Where only a composite or non-wooden club, or throwing stick, was mentioned for a given activity, this information was highlighted in the Dataset with an asterisk (*). In some cases, I was able to record the “sophistication” of the weapons—in other words, whether they were only “simple” unworked sticks or were “sophisticated” weapons with a special shape, decoration, and/or meaning. Other information recorded in the Dataset includes the material of weapons, the presence of composite and non-wooden clubs and throwing sticks, a list of other weapons and fishing gear used by the society (excluding firearms), SCCS identification, eHRAF identification, subregion, subsistence, and references.

The approach taken in this study does not strictly follow the proper methodology of cross-cultural research (Ember & Ember, 2009). Although I tried to prefer references corresponding to the SCCS (sources labelled S1, S2, and S3 in eHRAF), information from ethnographies that describe different groups or time periods were sometimes also included. Therefore, not all data strictly adhere to the focal time and place as defined in the SCCS. This should not be a problem for this study since the only other variable examined is subsistence, but those wishing to use the Dataset for correlations with other cultural features in future should treat it with caution.

Sometimes, it was unclear whether the *stick* was used as a blunt or sharp weapon. Unless authors specifically stated it was a “pointed stick,” “sharp stick,” or “speared with stick,” I assumed they meant a blunt weapon and therefore included it in the Dataset. Similarly, the material of the club, or throwing stick, was not always mentioned. In this case, it was coded as all-wooden since wood can be assumed to be the most common material for these types of weapons. In some cases, however, this assumption may be incorrect. At the same time, it is probable that not all authors have mentioned the use of all-wooden clubs in their works (e.g., when it was just a simple stick). For these reasons, the results may be slightly inaccurate. Nevertheless, the aim of this study is not to provide precise statistics, but rather to elucidate the variety of functions and main activities for which clubs and throwing sticks were known to be used.

### Results

Ethnohistoric and ethnographic sources provide evidence of clubs as contact weapons in 53 (93%) of the 57 foraging societies surveyed (see Dataset), including one society with the use of only clubs made of horn (Gross Ventre). Not counting this and two other societies for which the material of the club is not specified, a wooden club or stick is reported for 50 cases (88%). Only in the case of the Andamans was it explicitly stated that clubs were not in use (Man, 1932:142). However, Andamanese women occasionally fought with their wooden digging sticks (Radcliffe-Brown, 1922:43–44, 50). For Trumai there is conflicting evidence (Murphy & Quain, 1955:36), and for Barama River Carib and Vedda the evidence for clubs is missing from the ethnographic record, but their absence was not explicitly confirmed.

The use of throwing sticks/clubs was less frequent, recorded in 12 (21%) societies, including one society with only non-wooden throwing sticks (Maori) and one society with composite clubs used also for throwing (Creek). For six societies (Andamans, Eastern Apache, Nambicuara, Abipón, Tehuelche, Yahgan) the evidence for throwing sticks/clubs was ambiguous, and for 39 societies, I found no evidence. Since in many cases delivery methods were not specified, some of the contact clubs may also have been thrown at least occasionally.

**Fig. 1:**
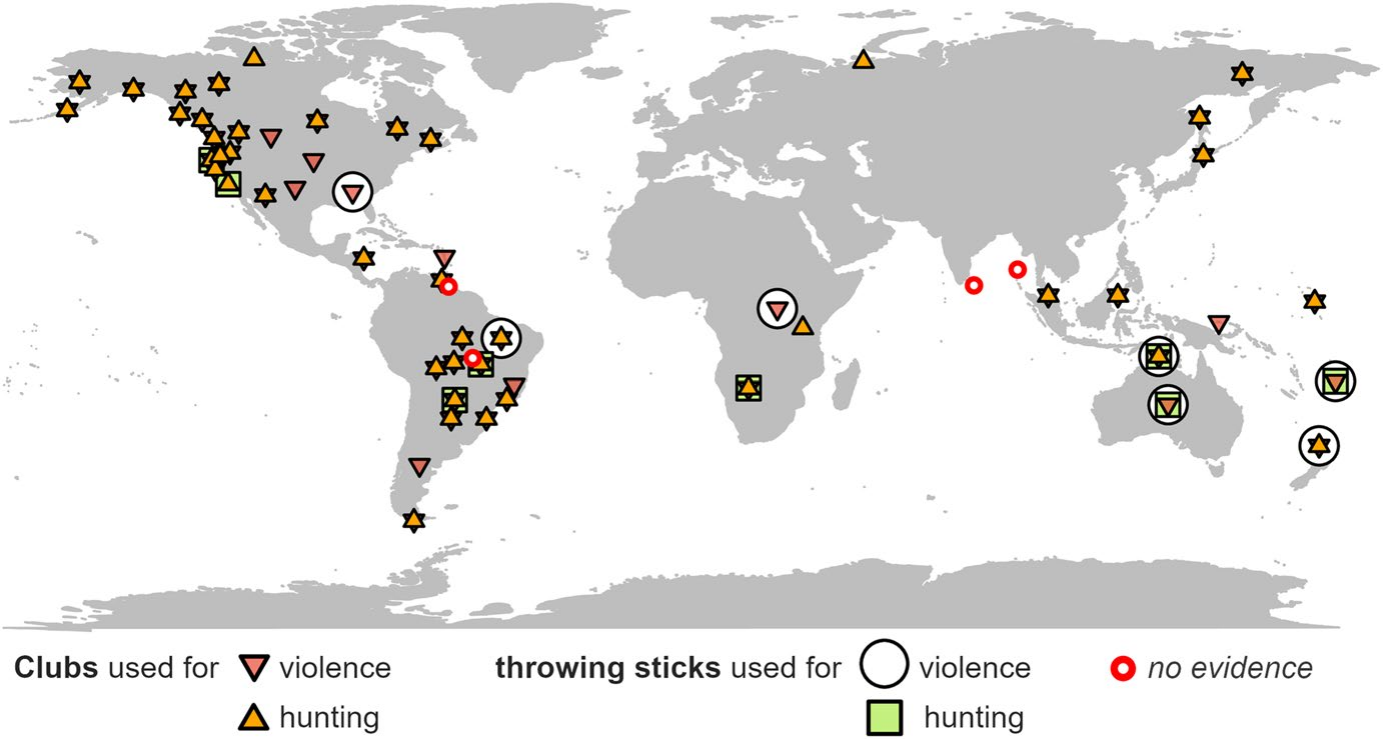
Global distribution of 57 foraging societies in the sample. Symbols indicate the documented use of clubs and throwing sticks (including non-wooden and composite weapons) for hunting and violence

The use of both clubs and throwing sticks is recorded for cultural groups in Africa, North and South America, and Oceania (Fig. 1). For Eurasian cultures, there is evidence only for contact clubs, but this may be due to the relatively small number of cases examined from this large area.

**Fig. 2:**
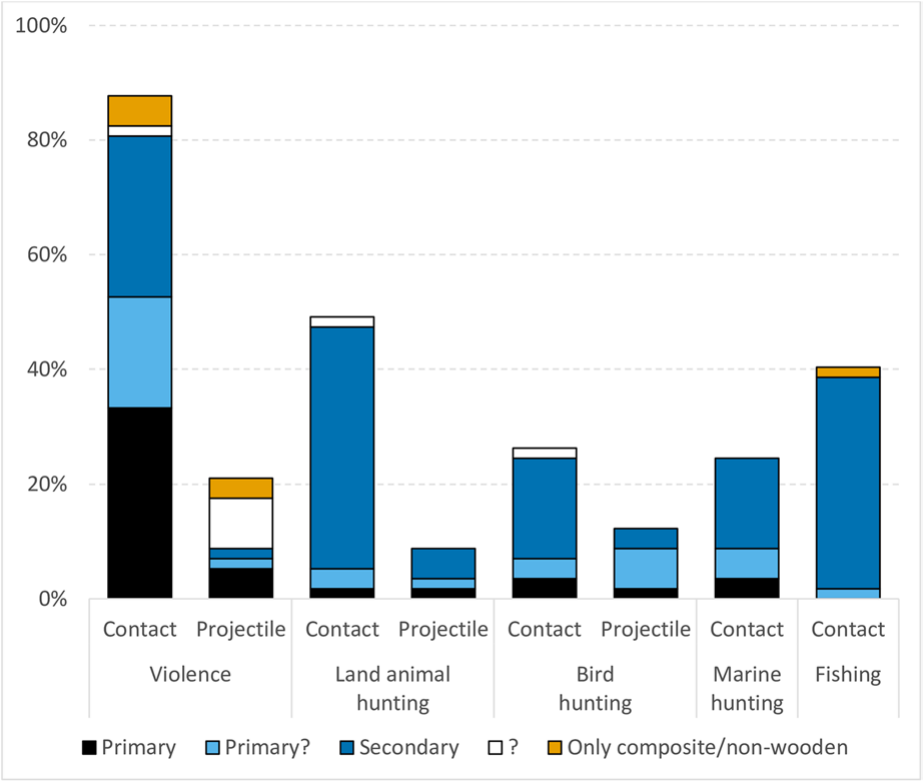
Five main activities for which wooden clubs (contact) and throwing sticks (projectile) were reported to have been used as a primary or secondary weapon (*n* = 57 societies)

Clubs were most commonly used for intergroup and interpersonal violence (Fig. 2, ESM Table S2). Thirty-three percent of societies used the club as the main or one of the main fighting weapons, whereas 28% used them only occasionally and for 19% the distinction between primary and secondary use was not possible. For one case, the use of clubs for violence was uncertain, and for three societies, only the use of composite and non-wooden clubs was documented. The second and third most common use of clubs was for hunting land animals (47%) and fishing (39%). For both of these activities, clubs were mostly used only as a secondary weapon. Only the African San used clubs (*knobkerries*) as one of their main hunting weapons (Lee, 1979:128–29). For both hunting marine mammals and hunting birds, 25% of societies used clubs among their weapons. Primary use of clubs was relatively rare, with only two cases in each category. However, for the other three and two cases, respectively, the frequency of use could not be distinguished and their role in these activities may also have been quite significant.

Where the club was used for hunting or fishing, it was usually also used for violence, although for each activity there might be a special type of club. This is not surprising given that violence was the most common function in the sample. However, there are a few exceptions (e.g., Yokuts, Copper Inuit).

Wooden throwing sticks were used both for violence (9%) and for hunting game (9%) and birds (12%; Fig. 2, ESM Table S3). On the contrary, there was no evidence of their use in fishing and marine hunting among societies surveyed, although fishing with a throwing stick is known from some Australian societies (Bordes, 2014:85–86). Only four societies (Aranda, Tiwi, Mbau Fijians, San) used throwing sticks as one of their primary weapons for violence and/or hunting. The former three (as well as Canela) used specialized throwing sticks/clubs, while San used multifunctional knobkerrie. For the remaining societies, the presence of specialized throwing weapons is not certain; they may have used multifunctional clubs or simple sticks. The act of throwing sticks or clubs for amusement was also recorded in a few cases (see Dataset).

The distribution of the recent use of clubs and throwing sticks by activity is shown in ESM Figs. S1–S5. Several observations can be made. The use of clubs for violence is documented in all regions where their presence is recorded (Fig. S1). The same is true for throwing sticks. The use of both weapons for hunting land animals is particularly lacking in South Asia and Oceania, except in Australia (Fig. S2). The use of clubs for marine hunting and fishing, unsurprisingly, occurs mainly in coastal societies (Figs. S4 and S5).

Although the presence of clubs is independent of the intensity of the foraging lifestyle (95% of hunter-gatherers [HG] and 89% of primarily hunter-gatherers [PHG]), differences in club use can be observed between the two sample groups (ESM Tables S4 and S5). The smallest differences are in the violence category, with PHG more likely to use wooden clubs as one of their main weapons (39%) compared with HG (31%). In contrast, the primary use of clubs for hunting land animals, birds, or marine mammals has been recorded only for HG societies. Overall, HG used clubs for all three hunting categories significantly more than PHG, roughly twice as often. In the fishing category, 44% of HG used clubs compared with 28% of PHG, with both types of society using them mostly as secondary weapons.

The use of throwing sticks for violence (including non-wooden and composite weapons) is more common in PHG (22%) compared with HG (8%), while for bird hunting the frequency of use is similar (about 12%), and for hunting land animals there is evidence only from HG societies (ESM Tables S6 and S7).

#### Violence

Wooden clubs were used for intergroup and/or interpersonal violence in at least 46 societies. In some cases, they were used only occasionally, but in 19 societies, war clubs formed an integral part of the weaponry (Fig. 2, ESM Table S2). In addition to wooden clubs, composite or non-wooden clubs (made of bone, horn or stone) were also, or exclusively, used for violence in at least 18 societies (see Dataset). The ways of using clubs in combat are unfortunately not described in all cases, but the use for close fighting seems to be prevalent. If the group also fought with bows, clubs were usually used after the supply of arrows was exhausted, as described for Enxet and Enlhet (Grubb, 1911:108), Island Carib (Rouse, 1948:559), and Tupinamba (Métraux, 1948:119–20). Among some Plains Indians of North America, only the very bravest warriors who wanted to win prestige fought hand to hand with clubs (Turney-High, 1941:87; Wallace & Hoebel, 1952:246). Similarly, in Fiji, even after the mid-nineteenth-century introduction of the musket, “the leading warriors still fought with clubs, and the desire of every young warrior was not to kill with a gun but with a club” since that was the only way to win knighthood (Tippett, 1968:76).

The use of throwing sticks/clubs for violence was recorded in seven societies. Tiwi (Basedow, 1913:300–1) as well as Bau Fijians (Tippett, 1968: Table 1) used several types of throwing sticks/clubs. Boomerangs were the characteristic weapon of the Aranda (Spencer & Gillen, 1927:521). Maori used whalebone throwing clubs (Buck, 1952:272). Canela had special throwing clubs and they sometimes also threw their two-edged sword clubs (Nimuendajú, 1946:153). Creek used their war clubs with metal points for both close range and throwing (Swanton, 1928:406). Mbuti sometimes throw sticks and stones at each other in anger (Turnbull, 1962:118–19). Moreover, some types of Bau Fijians’ clubs (Tippett, 1968: Table 1), as well as Canela’s two-edged sword clubs (Nimuendajú, 1946:153) and Kiribatti’s fighting clubs (Koch, 1986:248), were used for thrusting and stabbing.

Ethnographic sources give only occasional descriptions regarding clubs’ lifespans, ranging from the one-time use by Xokleng who “preferred to cut clubs from hard woods just before attacking and to throw them away when they were finished” (Henry, 1941:168) to long service life among Bau Fijians, among whom some war clubs continued to exist even after the death of their owner. As Tippett (1968:71–72) describes: “[After the chief's death] quite frequently his favorite club became the ‘shrine’ (waqawaqa) into which his ghost would enter to receive presentations from, or to communicate with the living.” Among Deg Xit'an (also known as Ingalik), “bone war clubs last until they crack, which depends on how much they are used. Wood clubs used in war are burned afterwards. ‘They have lots of blood on them.’ Sticks are discarded after being used” (Osgood, 1970:208). How often the same clubs were used for both hunting and warfare and how often specialized war clubs existed cannot be quantified precisely, but both possibilities were common.

The resolution of conflicts through duels with wooden clubs or staves was documented in a number of societies, including Ainu (Takakura, 1960:21–22), Botocudo (Métraux, 1946:536), Island Carib (Du Tertre, 1958:38), Nivkh (Black, 1973:28), and Slavey (Honigmann, 1946:86). Clubs were also used to punish crimes. The most frequently mentioned is adultery—for example, among Ainu (Takakura, 1960:20), Bau Fijians (Williams, 1860:22), Botocudo (Keane, 1884:206), Creek (Swanton, 1928:352–53), Island Carib (Rouse, 1948:556), Semang (Schebesta, 1954:253), and Xokleng (Henry, 1941:31). Other references are to punishing murder and sorcery—for example, Canela (Nimuendajú, 1946:159, 239–40) and Mundurucu (Murphy, 1958:106, 137). Tupinamba used ceremonial clubs to sacrifice prisoners (Métraux, 1948:122–24).

Other implements could also occasionally serve as a fighting club. In several societies, women used digging sticks as a weapon, while in others, people beat each other with paddles or bows (see Dataset).

#### Hunting Land Animals

**Fig. 3:**
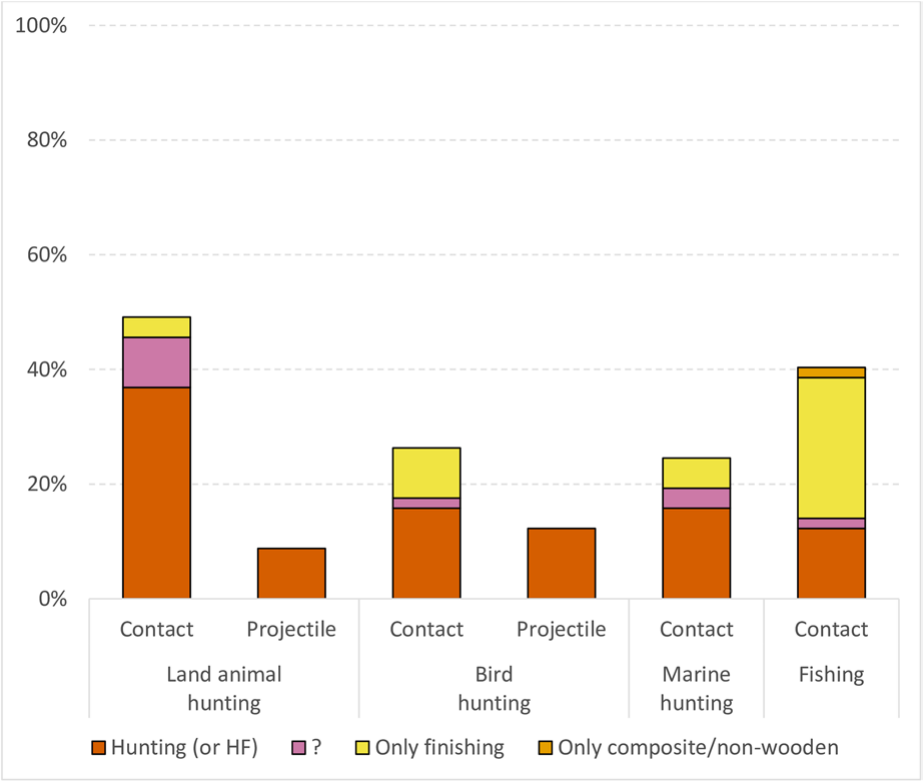
The proportion of societies in which wooden clubs (contact) and throwing sticks (projectile) were reported to have been used for hunting (i.e., catching and killing) or only for finishing off prey caught by other means. Divided by four main activities (*n* = 57 societies)

Using clubs to hunt land animals was recorded for 27 societies (47%). At least 21 of them used clubs for actual hunting, while two used clubs only for finishing off prey caught by other means. For the remaining four societies, the type of hunting was unclear (Fig. 3, ESM Tables S2 and S8). Clubs were used to hunt a relatively wide range of game. Of the more than 25 species recorded, the most frequently mentioned were armadillo, beaver, bear, deer, peccary, porcupine, and snake (Appendix).

Following prey size classes defined by Bunn (1982), the majority of prey hunted with wooden clubs fall into Size Class 1 (< 23 kg) and Size Class 2 (23–113 kg). Larger animals include moose (ca. 400 kg) and other big game reportedly hunted by Slavey (Asch, 1981:340), and, depending upon which specific species were hunted, possibly also bear reported for Ojibwa (Skinner, 1911:163–64), Kaska (Honigmann, 1954:36), and Pomo (Barrett, 1952:189–90), and alligator or caiman documented for Sirionó (Holmberg, 1950:26) and Tupinamba (Métraux, 1948:100).

Specific hunting strategies associated with clubs were not clearly described for all societies. In any case, clubs were found to be associated with all land-animal hunting strategies as defined by Churchill (1993), most often with disadvantage, pursuit, and encounter hunting (Table 1, ESM Table S9). Both hunting alone and communal hunting were described, the latter often in connection with disadvantage hunting—for example, to hunt deer (Elmendorf, 1960:93), wild pig (Heinen, 1972:135–36), rabbit (Opler, 1941:326; Wallace, 1978:450), or rodents (Métraux, 1948:100). Clubs were also reported to be utilized in conjunction with other technologies, including fire (Maybury-Lewis, 1967:42; Nimuendajú, 1946:65), dogs and ropes in deer hunting (Heinen, 1972:140; Watanabe, 1964:36), “bear tipi” (Honigmann, 1954:36), platforms in wolf hunting (Osgood, 1958:67–68), or ditches dug in rodent hunting (Métraux, 1948:100).

**Table 1:** Recorded combinations of techniques and delivery methods (clubbing/throwing) when hunting with clubs and throwing sticks

Hunting technique	Land animal hunting	Bird hunting	Marine hunting
Disadvantage	11 / –	5 / –	2 / –
Ambush	5 / 1	1 / 2	1 / –
Approach	3 / 1	4 / 1	8 / –
Pursuit	9 / 1	2 / –	– / –
Encounter	9 / 2	1 / 3	– / –

*Note*. *Disadvantage* refers to any technique that limits the escape of an animal. *Ambush* refers to instances in which hunters wait for prey in hiding, whether behind man-made blinds or natural features. *Approach* refers to stalking free-moving animals to within effective weapon range. *Pursuit* refers to chasing an animal to overtake it or to exhaust it. *Encounter* refers to hunting in which animals are taken, either jumped from the bush or spotted in trees, as they are encountered. For full definitions, see Churchill (1993)

The use of throwing sticks/clubs for hunting land animals was significantly less common. I found evidence for only five societies in the sample (Fig. 3, Appendix). For example, San used knobkerrie to knock small mammals such as mongoose (Lee, 1979:141); Tiwi used “their ironwood fighting clubs (*muraguŋa*) to down wallabies and flying foxes” (Goodale, 1971:160); and Aranda hunted “kangaroo, rock-wallabies, emus and other forms of game” with boomerangs (Spencer & Gillen, 1927:7).

#### Bird Hunting

Using clubs in bird hunting was recorded for 14 societies (25%). The ethnographies of nine of them mention use of clubs for actual hunting (Fig. 3, ESM Table S8). Only finishing off prey (e.g., caught in net or snare) is recorded for five societies. Two societies used clubs as one of their main weapons for hunting birds, namely Eyak (Birket-Smith & De Laguna, 1938:112–13) and Yahgan (Gusinde, 1960:228–46). In two other societies, the role of clubs is uncertain, but also potentially significant (Fig. 2, ESM Table S2).

For bird hunting, the use of throwing sticks was recorded in seven societies (12%), all for actual hunting (ESM Tables S3 and S8). Tiwi (Goodale, 1971:159) and Fijians (Tippett, 1968: Table 1) used special throwing sticks for hunting birds, while Aranda hunted birds with their boomerangs (Spencer & Gillen, 1927:17). For the others, the use of specialized throwing sticks is uncertain; they might have thrown multifunctional clubs, or just simple sticks.

Clubbing was particularly associated with disadvantage and approach hunting, whereas throwing projectiles mainly occurred during encounter and ambush hunting (Table 1, ESM Table S9). Although some societies could hunt birds with clubs and throwing sticks at any time, specific times of the year or day were emphasized for others. For example, Eyak killed birds chiefly in August when they were moulting (Birket-Smith & De Laguna, 1938:112), and Nivkh did likewise (Black, 1973:25–28). Enxet and Enlhet hunted birds with sticks during the wet season (Grubb, 1911:83–84). The latter also hunt birds at dusk, like Tiwi (Goodale, 1971:159–60), or at night using a torch to blind them, like Yahgan (Gusinde, 1960:233, 245) and Mi'kmaq (Denys, 1908:435–36). Communal bird hunting was recorded, for example, for Eyak (Birket-Smith & De Laguna, 1938:112–13), Enxet and Enlhet (Grubb, 1911:83–84), and Northern Paiute (Kelly, 1932:90). For the list of specific birds reportedly hunted with clubs and throwing sticks, see the Appendix.

#### Marine Hunting

Using clubs to hunt marine mammals was recorded for 14 societies (25%). Nine of them used clubs for actual hunting, whereas three societies used clubs only for finishing off prey caught by other means (e.g., harpoon or bow). For two societies, the type of hunting was unclear (Fig. 3, ESM Table S8). As with bird hunting, the two societies that used clubs as one of their primary weapons were Eyak (Birket-Smith & De Laguna, 1938:107) and Yahgan (Gusinde, 1960:155, 217–22). In three other societies, the role of clubs is uncertain, but also potentially significant (Fig. 2, ESM Table S2).

The marine animal most frequently hunted with clubs was seal, followed by sea lion and sea otter (Appendix). By far the most common strategy was approach hunting; other hunting tactics were recorded less frequently (Table 1, ESM Table S9). Seal and sea lion were most often killed with clubs while sunning and sleeping, as documented, for example, for Nivkh (Schrenck, 1964:849), Innu (Lane, 1952:8), Pomo (Barrett, 1952:189), Southern Coast Salish (Smith, 1940:254), and Yahgan (Gusinde, 1960:218–19). Special hunting tactics included the use of decoys and disguises in seal hunting reported for Mi'kmaq (Wallis & Wallis, 1955:29–30) and Aleutian sea otter hunting during severe storms (Veniaminov, 1840:344–45). There is no mention of marine hunting with throwing sticks for any of the analyzed societies; animals were always clubbed at close range (for a possible exception see Gusinde, 1960:218, n.232). Communal marine hunting was recorded rather rarely—for example, among Aleut (Veniaminov, 1840:362–63).

### Fishing

In 22 societies (39% of the sample), almost all of them used the club in fishing only as a secondary weapon (Fig. 2, ESM Table S2). Only among Kaska, who used a foot-long birch or poplar pole to club fish as they swam in shallow water, was there a more significant role. However, even they used the stick/club only seasonally in periods of open water (Honigmann, 1954:37–38).

Compared with its use in other types of hunting, the club was mainly used to finish off fish caught in other ways (e.g., hooked, speared, netted, and poisoned). Only seven societies used the club for actual catching of fish (Fig. 3, ESM Table S8). Like the Kaska, they fished with sticks/clubs only in shallow waters—for example, at low tide (Koch, 1986:16–17), in the shallows near the shore in winter (Chamberlain, 1892:565), in small ponds and streams which dry up in the dry season (Holmberg, 1950:27), at small rapids during the spring (Rogers & Black, 1976:7), or by fencing off a small creek or narrow brook (Gusinde, 1960:273; Heinen, 1972:136–37).

Finally, some societies had special clubs for fishing, such as the “salmon knocker” among Southern Coast Salish (Elmendorf, 1960:83), “sturgeon mallet” among Ojibwa (Skinner, 1911:137), *isabakiku* for salmon fishing among Ainu (Takakura, 1960:13), *takū* for killing large fish including shark among Kiribati (Koch, 1986:11), or “special knob-headed fish club” among Pomo (Barrett, 1952:153).

## Discussion

### Indirect Evidence for the Existence of Clubs and Throwing Sticks in the Pleistocene

How can we be fairly certain that early humans used wooden clubs and/or throwing sticks as their weapons, when archaeological evidence from the Pleistocene is so rare and the original function of the found artifacts is difficult to prove? One line of evidence is the widespread distribution of clubs among hunter-gatherers, as shown by the analysis above. However, this argument is not without problems. Recent foragers are in many ways specific, and their lifeways cannot be simply projected back into the past (Ember & Ember, 1995; French, 2019; Kelly, 2013: chap. 10; Marlowe, 2005). Therefore, it is necessary to consider other indirect evidence, such as the universal cross-cultural use of sticks by young children at play (Edwards, 2000; Ember & Cunnar, 2015). One can easily imagine these toys being transformed into tools and weapons in adulthood (see Milks, 2020 for wooden spears).

Another supporting argument for why early *Homo* *sapiens* may have used clubs and throwing sticks comes from primatology. Of all the modern nonhuman primates, the use of sticks as throwing and clubbing weapons has been observed most in chimpanzees (Shumaker et al., 2011). Wild chimpanzees were seen throwing sticks and branches, as well as other objects, at conspecifics, humans, baboons, monkeys, leopards, and lions (Boesch & Boesch, 1989; Goodall, 1964, 1986; Van Lawick-Goodall, 1968). Dragging, waving, and unaimed throwing of branches are also typical for agonistic charging displays of male chimpanzees. Play-throwing was seen in infants and occasionally in juveniles and adolescents. Clubbing was reported to be directed at other chimpanzees, but also at humans and other animals such as leopards, baboons, snakes, and insects (Boesch, 1991; Goodall, 1986; Pruetz et al., 2017; Van Lawick-Goodall, 1968; Watts, 2008; Whiten et al., 2001). At the Fongoli site in Senegal, chimpanzees have even been observed hunting bushbabies with modified sticks used as “spears” (Pruetz & Bertolani, 2007).

The use of wooden weapons is not limited to chimpanzees; it has been observed in other great apes and monkeys as well (Shumaker et al., 2011). For example, white-faced capuchins were observed to use a branch as a club to attack a venomous snake, to hit each another with a stick, as well as to throw sticks and branches at other animals, such as coatis, tayras, opossums, spider monkeys, and peccaries (Boinski, 1988; Chevalier-Skolnikoff, 1990). Mannu and Ottoni (2009) described two incidents in which wild bearded capuchin monkeys attempted to club an opossum and a scorpion with a stick. Wild orangutans were observed breaking off branches and dropping or throwing them toward humans, other orangutans, and other species (Galdikas, 1982; Mackinnon, 1974; Schaller, 1961). At Tanjung Puting National Park, orangutans spontaneously hit conspecifics, humans, dogs, or monkeys with sticks (Galdikas, 1982); Kaja Island orangutans used a branch to club fish in shallow water and then caught and consumed them (Schuster et al., 2008:110, 128). Gorillas throw branches and twigs while displaying in the presence of conspecifics and humans, and they occasionally use branches and sticks for clubbing (Shumaker et al., 2011:129–31). Bonobos use sticks and branches to throw at and hit conspecifics, humans, and birds in agonistic and playful contexts (Gold, 2002; Jordan, 1982). Kano (1979) reported that wild bonobos cornered a small duiker and hit it with branches; they played with the duiker but did not kill or eat it.

Most of this evidence is rare. Aimed throwing and clubbing is not customary even among chimpanzees. Comparing nine African study sites, Whiten et al. (1999, 2001) showed that although dragging a large branch in display is universal, using it for hitting as a club is not. While the behavior was present or even habitual (i.e., occurred repeatedly in several individuals) in some chimpanzee groups, it was not observed in others. It also seems that sticks and branches are only picked up in a given location where they are immediately used. Since they are ubiquitous, they are not transported over long distances. As Boesch and Boesch (1984) observed, wooden clubs used for cracking nuts are regularly transported for less than 20 m (in contrast to stone hammers, which are moved up to 500 m). Moreover, although brandishing sticks, like throwing stones, is an extremely effective method of intimidation, these weapons are seldom used in serious fighting. Chimpanzees prefer biting, hitting, and stamping (Goodall, 1986:551–52).

To conclude, the presence of wooden clubs and throwing sticks in the Pleistocene cannot be proven without new direct archaeological evidence. However, based on the indirect evidence from recent hunter-gatherers and nonhuman primates, at least the occasional use of crude sticks for throwing and hitting is highly probable (see also Marlowe, 2005; Bordes, 2020). The second question is how sophisticated these early weapons were and how significant their role was in everyday life.

### Functions and Forms of Clubs

*Homo* *sapiens* and *Homo* *neanderthalensis* differ from other primates in their ability to create much more sophisticated tools and weapons. That also applies to clubs. Although even recent foragers used any branch or log which happened to be at hand as their weapon, in more than half of the societies the clubs were specially shaped, decorated, and/or composed of several parts (see Dataset). The wide variability of clubs demonstrated from several regions (e.g., Cowper, 1906:49–60; Taylor, 2001:16–29) shows that this weapon was more than just a mere stick, and its production was certainly not as simple as it may seem at first glance (for wooden spears, see Haidle, 2009).

A perfect illustration of this is Fiji, where some of the greatest variation in clubs has been documented (Tippett, 1968). In addition to war clubs, which included prodders/stabbers, strikers, penetrators, and throwers, there were peacetime, ceremonial, and sacred clubs. Each of these types was further divided into several subtypes. Manufacture of clubs was a highly developed industry and of great importance to Fijian society. Diversification was greatest in the war clubs, and no other craft was more open for experimentation. Some Fijian clubs required years to create. As Tippett (1968:50–52) points out:

The craftsman had to know the natural environment and its vegetation. The maker of clubs had to recognize the potential of every tree—the root, the straight limb, the forked limb. He had to know the woods. Much of his work was done while the tree was yet growing. For months, sometimes for years, he would work on the *waka*, or roots of a selected tree before uprooting it. He knew the type of carving required on each wood, which types ought to be carved when still wet, and which were to be buried for a time in the mangrove swamp before working. The natural environment limited him, but it also tested his ingenuity and developed his inventiveness. . . . It was not any man who could make a club—even a *vunikau*, the simplest rootstock. Experience was more than knowing the resources of the environment, although it did include this; for the craftsman drew from the fellowship of other club makers and the accumulated knowledge of the craft group handed down from past generations.

Clubs did not have to be mere weapons but could also carry a strong symbolic meaning. Among the Shavante, for instance, each of the three major types of club was associated with (but not necessarily used exclusively by) one of the age-grades (Maybury-Lewis, 1967:242). Over time, the club transformed into other composite or non-wooden striking weapons, which often took on the functions of symbols and insignias of power and reign—for example, ceremonial mace, bulava, royal scepter, marshal’s baton, and swagger stick (Vencl, 1979).

### Clubs Compared with Other Weapons

As cross-cultural comparison shows, the wooden club is a multifunctional, primarily close-range or contact weapon, used for hunting, fowling, fishing, duels, warfare, and amusement. Compared with projectile weapons, however, it has several significant disadvantages. Although it is possible to safely club small animals, close confrontation with any medium-sized or large animal is always risky. One well-aimed kick or impact with the animal’s horns can cause serious, potentially fatal injury. Rodeo athletes with their frequent fractures illustrate the danger of such close contacts with large animals (Berger & Trinkaus, 1995). At the same time, the need to get close increases the chance that the prey will spot the hunter and run away. Since many projectile weapons can be archaeologically dated back to the Middle Pleistocene—for example, spears to 500,000–300,000 BP (Allington-Jones, 2015; Schoch et al., 2015; Wilkins et al., 2012) —it can be argued that they were preferred over contact clubs.

Contrary to previous assumptions (e.g., Churchill, 1993), Milks (2020) demonstrated on the basis of ethnographic evidence that even untipped wooden spears “are not limited to either small or large game procurement, and are capable of killing a variety of animals of different size classes and with differing behaviours and ecologies” (2020:11). They can serve as contact thrusting weapons, as hand-thrown weapons, or as multifunctional implements, both in hunting and interpersonal violence. The ethnographic data demonstrate the capability of both lightweight and heavy wooden spears to be thrown at considerable distances of up to 50 m (Milks et al., 2019: supplementary information). Innovation in the form of composite spears tipped with stone, bone, or other materials may have further improved lethality of the weapon and thus potentially affected the frequency and regularity of hunting success (Salem & Churchill, 2016; Wilkins et al., 2014).

“Complex” or mechanically projected weapons such as the spear-thrower and dart, or the bow and arrow, also have a long history. Based on the proxy evidence of stone points, they are dated to at least 80,000–70,000 BP (Lombard & Shea, 2021). Compared with hand-thrown spears, complex projectiles likely have a higher accuracy at distance, require less power to launch, and hitting a target with them is a relatively easy skill to learn (Milks et al., 2019). Ethnographic evidence and experimental studies show that both spear-throwers (atlatls) and bows are very effective weapons in hunting practically all types of game, from small rodents and birds to elephants (Cattelain, 1997; Churchill, 1993; Hutchings & Brüchert, 1997; Tomka, 2013).

The development of complex weapons may have led to a decline in the use of clubs, but it certainly did not lead to their complete abandonment. Like wooden spears (Milks, 2020), clubs have been continually used alongside other types of weapons up to the present. Written sources attest to the use of wooden clubs from both ancient Mediterranean civilizations and the European Middle Ages (for an overview see: Jähns, 1899:156–61; Kontny, 2015:275–77; Vencl, 1979:651–52). Many Indigenous societies of North America, including those subsisting on agriculture, used wooden clubs not only in warfare and hunting, but also in ceremonial contexts (Taylor, 2001:16–29). The pastoral Tuareg traditionally used wooden clubs for many different purposes, including cattle-driving, hunting birds and small animals, killing poisonous snakes, and personal protection (Nicolaisen, 1963:170: Fig. 120). Wooden baseball bats are still a “silent” weapon in the present-day United States (Dujovny et al., 2009).

Ethnographic and historical accounts show that wooden clubs can serve as hunting tools, but their main use is probably for intergroup and interpersonal violence. There are at least two reasons for their use in combat, which distinguish them from other weapons. First, they may be preferred in skirmishes where the primary objective is not to kill the enemy. An example are South American Yanomamo, who had several forms of aggressive activities. While bows and arrows were used for actual fighting and killing, dueling with 2- to 3-m-long clubs was among the most “innocuous” form of violence, along with chest pounding and side slapping. Even so, club fights could sometimes quickly escalate into bloodshed (Asch & Chagnon, 1975; Chagnon, 1977:118–20). In addition to the foraging societies cited above, ritualized duels with sticks/clubs can be found among the hunter-gatherer Ache of Paraguay (Hill & Hurtado, 1996:70–73) and also African agropastoral Surma (Abbink, 1999) and Nuer (Evans-Pritchard, 1940:151). Second, traditional warfare is not just about defeating the enemy, but also about courage and heroism. A brave warrior cannot become a hero by cowardly shooting, but by confronting the enemy and defeating him in a personal hand-to-hand duel, for example, with a war club (Tippett, 1968:76; Turney-High, 1941:87; Wallace & Hoebel, 1952:246).

### The Role of Throwing Sticks

Throwing sticks exist in a wide variety of forms and can be used not only as projectiles but also as contact weapons and tools (Bordes, 2014; Davidson, 1936). Ethnographic and historical records document their multifunctional use for hunting land animals (including large game such as kangaroos, deer, buffalo, and emu), hunting birds and bats, fishing, fighting close and at a distance, parrying, herding, fire management, and amusement. They also had a symbolic and social functions, being used as musical instruments, parts of dance costumes, ceremonial objects, or as a currency of exchange (Bordes, 2014:85–96).

Although made of the same perishable materials as the clubs, throwing sticks are more likely to be recognized in the archaeological record due to the fact that many of them had characteristic curved shapes and/or pointed ends, such as those from Schöningen (Conard et al., 2020; Thieme, 1997) and Oblazowa (Valde-Nowak et al., 1987). Compared with non-throwing clubs, they had the advantage of being used at a distance, making them safer and more effective weapons (Bordes, 2014; Bordes et al., 2015). However, whether they were more widespread among prehistoric humans remains unknown.

As shown above, cross-cultural distribution of throwing sticks among recent foragers is much smaller than that of striking clubs (Fig. 1). This can probably be explained by the presence of mechanically projected weapons in most recent hunter-gatherer societies. Although “throwing sticks were efficient weapons and should not be underestimated when compared to spear and arrow damage” (Bordes, 2020), they were nevertheless “more adapted to use in open woodland and dry savanna without too many obstacles and on levelled ground” (Bordes, 2020). In particular, the versatility of the bow, along with its high accuracy and fast learning curve (Cattelain, 1997; Whittaker, 2013; Yu, 2006), may have led to the replacement of the throwing stick as the main projectile weapon and its gradual abandonment (Bordes, 2014:54). Although examples of societies that simultaneously used multiple projectile weapons are known, highly mobile hunter-gatherers may have reduced the number of weapons to minimize their burden during their frequent moves through the country. Therefore, stick throwing, as well as stone throwing (Isaac, 1987; Wilson et al., 2016), could have been much more common and had a wider application in periods before the inventions of the bow and spear-thrower. On the other hand, for throwing sticks to function as an effective projectile weapon, real technical skill is needed for their construction and especially to throw them properly (Bordes, 2014:19). Coupled with their limited effectiveness in some types of environments, the small cross-cultural distribution of throwing sticks may also suggest that they have never become a regular part of weaponry in many cultural areas.

## Conclusion

The absence of distinctive and durable components that are part of other weapons, such as spears, arrows, maces, or axes, means that only very few Pleistocene wooden clubs and throwing sticks have been preserved until the present day. The quantitative analysis of recent foragers, together with other arguments presented in the discussion section, clearly shows that the use of these weapons by early humans was not only possible, but also highly probable, despite the lack of direct archaeological evidence.

Although the club may have served only as a secondary tool in hunting and fishing, in combat it may have been an integral weapon of any prehistoric warrior. This is not to say that all Pleistocene human groups used clubs with equal intensity. As with recent cases, we can assume that the use of clubs varied from society to society, with some using them more, others less, and some perhaps not at all. It can also be assumed that the prehistoric club was not some standardized piece of weaponry. On the contrary, it could take many forms from a simple crude stick to a long stave to a curved and decorated heavy club with a strong symbolic meaning, which existed only in certain regions or periods.

The prehistoric use of throwing sticks is more difficult to assess. Throwing sticks (including specialized projectiles such as boomerangs) for hunting and violence undoubtedly existed long ago in the human past, as evidenced by archaeological finds and iconographic depictions. On the other hand, the cross-cultural evidence for sophisticated throwing sticks (beyond the simplest form of a crude stick) concentrates only on certain regions, which may indicate that a similarly limited distribution may have existed in the past.

As far as the issue of simplicity is concerned, one should not throw the baby out with the bathwater. The fact that Neanderthals and early *Homo* *sapiens* were capable of creating complex tools should not mean we erase from their lives anything that smacks of simplicity—in this case, clubs. An equivalence that simple technologies reflect inferior intellects is false, but so is the opposite. Great cognitive abilities do not mean absence of relatively simple technologies.

Preference for different types of weapons depends on a combination of many factors, including the environment (terrain, vegetation, availability of raw materials), availability of game, its type and behavior, the hunter’s skills, and cultural traditions. Variations in hunting and warfare tactics therefore could have kept clubs and throwing sticks effective and useful, even after the introduction of other “more complex” weapons.

Finally, this study shows that even the unsystematic use of ethnographic analogies carried out more than 150 years ago (Lubbock, 1865:475) can lead to the right conclusions—the antiquity of the wooden club and its important role in the past—albeit a more sophisticated weapon than earlier authors imagined.

### Electronic supplementary material

Below is the link to the electronic supplementary material.

Supplementary file1 (PDF 1235 KB)

## Data Availability

The data for this manuscript are available on the Open Science Framework at https://osf.io/wydsj.

## Appendix

Prey hunted (H) and/or finished off (F) with clubs/sticks (contact) and throwing sticks (projectile).

**Table:**

Society	Land animal hunting		Bird hunting		Marine hunting	Fishing
	Contact	Projectile	Contact	Projectile	Contact	Contact
San	Porcupine, ant bear, snakes, cheetah, immature animals (H); Antelope, steenbok, gemsbok, springhare, duiker (F)	Mongoose, steenbok, duiker, squirrel, hare and other small animals (H)	Birds (H)	Korhaan, guinea fowl and other birds (H)		
	(?) Honey badger (H)	(?) Honey badger (H)				
Hadza	unspecified animals (?)					
Nenets			Goose (F)			
Semang	Monkey (F)					
Sama-Bajau						Shark (F)
Tiwi	Goanna, snake (H)	Flying fox (H)		Goose (H)		
	Wallaby, possum (H)	Wallaby, possum (H)				
Aranda		Kangaroo, rock-wallaby, emu, etc. (H)		Pigeon, eagle-hawk, goose, brushturkey, pheasant, duck, cockatoo etc. (H)		
M Fijians				Pigeon and other birds (H)		
Maori			Birds (F)			Eel and other fish (F)
Kiribati						Eel (H); Shark and other large fish (F)
Ainu	Deer (F)				Seals (?)	Salmon (?); Fish (F)
Nivkh	Otter, rat (H)		Birds (H)		Sea lion, seal (H)	Sturgeon, hausen (H, F)*
Yukaghir	Fox, arctic fox (H)					
Ingalik	Fox, wolf, porcupine (H); Beaver (F); Lynx (?)					Fish (F)
Aleut					Sea otter, sea lion (H); Seal (H, F)	Halibut and other fish (F)
Copper Inuit					Seal (F)	Arctic char, tomcod, trout, etc. (F)
Innu	Beaver (H, F); Porcupine (H); Moose (F)				Seal (H)	
Mi'kmaq	Beaver (H)		Goose, Brant, Duck (H)		Seal (H)	
Ojibwa	Bear (H); Small animals (?)		Duck, swan and other birds (H)			Walleye, sucker (H); Sturgeon and other fish (F)
Slavey	Moose and other big game (H?, F); Beaver, trapped animals (F)					
Kaska	Porcupine, bear (H); Beaver, marten, trapped animals (F)					Fish (H)
Eyak			Duck, goose, swan etc. (H)		Seal (H)	Salmon, halibut (F)
Haida					Sea otter, seal, sea lion (F)	Halibut (F)
Nuxalk	Marten (H)				Seal (H, F)	
SC Salish	Deer (H); Bear (F)		Duck (F)		Seal (H, F); Porpoise (F)*	Salmon and other large fish (F)
Yurok		small game (H)		Duck (H)	Sea lion (H?)	Salmon (F)
Pomo	Bear, ground squirrel (H)		Water birds (F)		Seal, sea lion (H)	Fish (F)
Yokuts	Rabbit, cottontail, squirrel (H)	Rabbit, cottontail, squirrel (H)				
N Paiute	Bear (H); (?) Deer, groundhog (F)		Sage hen, mud hen, goose (H)			Fish (F)
Klamath			Duck and other water birds (F)			Fish (F)
Kutenai			(?) Fool hen (H)			Fish (H)
E Apache	Rabbit, wood rat (H)		Eagle (H, F); Turkey (?)			
Miskito					Manatee (F)	Snook, tarpon and other large fish (F)
Warao	Wild pig (H); Armadillo (H?); Deer (F)					Fish (H, F)
Mundurucu						Fish (F)
Sirionó	Peccary, alligator/caiman, coati, anteater, armadillo (H); wounded animals (F)					Fish (H)
Nambicuara	Snake (H)					
Canela	Wild pig, anteater, armadillo, anaconda and other snakes (H)					
Tupinamba	Rodents, sloth (H) Caiman (F)					
Xavante	unspecified animals (H)			Macaw (H)		
Xokleng	Brazilian deer, collared peccary (H)					
E & E			Waterfowl (H)	Jabiru (H)		
Abipón	unspecified animals (?)					
Yahgan			Penguin, steamer duck (H); Cormorant (H, F); Wild goose (F)		Seal, sea lion (H, F)	Fish (H)

*Note*. M Fijians = Mbau Fijians; SC Salish = Southern Coast Salish; N Paiute = North Paiute; E Apache = Eastern Apache; E & E = Enxet and Enlhet. * = only a composite or non-wooden club recorded. For marine hunting and fishing, ethnographic sources only mention the use of contact clubs. For references, see the Dataset at https://osf.io/wydsj.

## Footnotes

1 Since the end of the nineteenth century, the archetype of the caveman with a club has appeared in literature (e.g., Stanley Waterloo’s *The* *Story* *of* *Ab* from 1897, Jean M. Auel’s *The* *Clan* *of* *the* *Cave* *Bear* from 1980), comic book series (e.g., *Alley* *Oop* since 1932, Vo'houna since 2002), cartoon series (e.g., *The* *Flintstones* since 1960, *Captain* *Caveman* *and* *the* *Teen* *Angels* since 1977), movies (e.g., *Quest* *for* *Fire* from 1981, *RRRrrrr!!!* from 2004), computer games (*Prehistorik* from 1991, *Caveman* *Tales* from 2020), toys (e.g., LEGO Caveman & Cavewoman from 2017, Cave Club doll series by Mattel from 2020), etc. See also Laskow (2015).

2 Published on January 13, 2020; available at https://gimletmedia.com/shows/every-little-thing/8whdexw

3 Throwing sticks were probably completely missing from the first visual reconstructions of prehistoric people; I have found only one possible throwing club in the scene of the giant moa hunting in the book *La* *Création* *de* *L'Homme* *et* *les* *Premiers* *Ages* *de* *Humanité* (Cleuziou, 1887:29).

4 https://ehrafworldcultures.yale.edu/
