# Supplementary material for: The Use of Wooden Clubs and Throwing Sticks among Recent Foragers: Cross-Cultural Survey and Implications for Research on Prehistoric Weaponry
Source: Hum Nat. 2023 Mar 29;34(1):122–52. doi: 10.1007/s12110-023-09445-3 (PMC10073058; doi:10.1007/s12110-023-09445-3)

Supplementary Information

Article: **The Use of Wooden Clubs and Throwing Sticks among Recent Foragers: Cross-Cultural Survey and Implications for Research on Prehistoric Weaponry**

Author: Václav Hrnčíř[1,2]

Affiliations: [1] Max Planck Institute for Evolutionary Anthropology, Department of Linguistic and Cultural Evolution, Leipzig, Germany
[2] Czech Academy of Sciences, Institute of Archaeology, Prague, Czechia

Contact: hrncir.vaclav@gmail.com

Journal: Human Nature

Table S1 Artistic reconstructions of Pleistocene life published between 1800 and 1950. Only depictions of prehistoric people with weapons/tools included.

Year	Title	Species (based on fossils from site / period)	Artist	Scientist /Author	Original publication	Reprinted in	Weapons/Tools
1838	L'Homme Fossile (*Fossil Man*)	*unspecified pre-sapient* (partly inspired by fossils from Schmerling Caves, Belgium)	Johann C. Susemihl	Pierre Boitard	Magasin Universel (April 1838)	Rudwick 2008: 420	Stone axe
1846	Homo Hercules Columarum (*Pillars of Hercules Man*)	today H. neanderthalensis (Forbes' Quarry, Gibraltar)	Thomas H. Huxley	Thomas H. Huxley	*unpublished sketch*	Ruddick 2009: 32	Stone axe
1851	Periode der Jetzwelt (*The Period of the Present World*)	H. sapiens (Adam and Eve)	Joseph Kuwasseg	Franz Unger	*Die Urwelt in ihren verschiedenen Bildungsperioden (1847, pl. XIV)*	Moser 1998: 118	**Stick or wooden club (?)**
1861	L'Homme Fossile (*Fossil Man*)	*unspecified pre-sapient* (partly inspired by fossils from Schmerling Caves, Belgium)	Pierre Boitard (engraved by Gustave Moreau)	Pierre Boitard	Paris Avant Les Hommes (1861, cover)	Moser 1998: 135<br>Pettit & White 2011: 27<br>Ruddick 2009: 19	Stone axe
	Période Anthropique: Dernier Age Paléontologique – Apparition de L'Homme (*Anthropic Period; Last Paleontological Age: Appearance of Man*)	*unspecified – probably H. sapiens*	Pierre Boitard (engraved by Bisson & Cottard)	Pierre Boitard	Paris Avant Les Hommes (1861, p. 239)	Moser 1998: 137	Stone axe, bow
1863	Apparition de L'Homme (*Appearance of Man*)	H. sapiens (Adam and Eve)	Edouard Riou	Louis Figuier	La Terre Avant le Deluge, 1st edition (1863, p. 363)	Moser 1998: 120<br>Pettit & White 2011: 28	**Stick (?)**
1866	Apparition de L'Homme (*Appearance of Man*)	*unspecified – probably H. sapiens*	Edouard Riou	Louis Figuier	La Terre Avant le Deluge, 5th edition (1866, p. 427)	Moser 1998: 123	Stone axe
1870	Une famille à l'âge de pierre (*A Family of the Stone Age*)	*unspecified – probably H. sapiens*	Emile Bayard	Louis Figuier	L'Homme Primitif (1870, cover)		Stone axe, **wooden club (?)**
	L'homme a l'epoque du grand ours et du mammouth (*Man in the Great Bear and Mammoth Epoch*)	*unspecified – probably H. sapiens*	Emile Bayard	Louis Figuier	L'Homme Primitif (1870, p. 53)	Moser 1998: 127	Stone axe, **wooden club**
1873	The Neanderthal Man	H. neanderthalensis (Neandertal valley, Germany)	Ernest Griset	?	Harper's Weekly (July 1873)	Moser 1998: 138<br>Sommer 2006: 225<br>Bermann 1999: 289	Stone axe, stone-tipped spear
1885	La fuite devant le mammouth (*Escaping from the Mammoth*)	*Unspecified (Magdalenian period)*	Paul Jamin	?	*oil on canvas* (Salon de 1885, Paris, France)	Capitan 1903: plate II	Spear, stone mace, dagger (?)
1886	Les Premiers Ages de L'Humanite: Epoque de l'ours des Cavernes (*The Early Ages of Humanity: Age of the Cave Bear*)	*unspecified – probably H. sapiens*	S. de Drée	Camille Flammarion	Le Monde Avant la Création de L'Homme (1886, plate V)		Stone axe (?), spear (?), **wooden club (?),** rock (?)
1887	Un drame à l'âge de pierre (*A drama in the Stone Age*)	*unspecified* (*Acheulean period*)	Paul Jamin	?	*oil on canvas* (Salon de 1885, Paris, France)	Capitan 1903: plate III	Spear, stone axe
1887	Ils disputaient leurs vies aux bêtes féroces (*They risk their lives with ferocious beasts*)	*unspecified – probably H. neanderthalensis or sapiens*	E. A. Tilly	Henri du Cleuziou	La Création de L'Homme et les Premiers Ages de Humanité (1887, p. 5)	Moser 1998: 131	Stone axe, stone-tipped spear

1887	Le Dinornis oiseau contemporain de l'homme primitive (*The Dinornis bird contemporary of the primitive man*)	*unspecified – probably H. sapiens*	?	Henri du Cleuziou	La Création de L'Homme et les Premiers Ages de Humanité (1887, p. 29)		Sling or **throwing club (?)**
	L'homme de l'epoque chelléenne (*The Man of the Chellean Period*)	*unspecified* (*Chellean period*)	Georges Devy	Henri du Cleuziou	La Création de L'Homme et les Premiers Ages de Humanité (1887, p. 165)		Stone axe, **wooden club (?)**
	L'homme vainqueur de l'ours des cavernes (*Man the Conqueror of the Cave Bear*)	*unspecified* (*Mousterian period*)	Georges Devy	Henri du Cleuziou	La Création de L'Homme et les Premiers Ages de Humanité (1887, p. 193)	Moser 1998: 132	Stone dagger
	Reconstitution de l'homme du Moustier (Reconstruction of the Moustier man)	*unspecified* (*Mousterian period*)	E. A. Tilly	Henri du Cleuziou	La Création de L'Homme et les Premiers Ages de Humanité (1887, p. 204)		Stone dagger, spear, **wooden club**
1894	*without title*	*unspecified – probably H. sapiens*	Worthington G. Smith	Worthington G. Smith	Man the Primeval Savage (1894, frontispiece)	Pettit & White 2011: 37 Moser 1998: xix	**Wooden club**, hand axe
1986	An eviction scene at Wookey Hole, near Wells - Older Stone Age	*unspecified* (tools from Wookey Hole, UK)	Cecil Aldin	Henry N. Hutchinson	Prehistoric Man and Beast (1896, frontispiece)	Moser 1998: 141	Bone-tipped harpoon
	Hunting the mammoth in southern France	*unspecified* *(Older Stone Age)*	Cecil Aldin	Henry N. Hutchinson	Prehistoric Man and Beast (1896, plate II)	Moser 1998: 142	Harpoon or spear, rock
	Hunting the reindeer in southern France	*unspecified* *(Older Stone Age)*	Cecil Aldin	Henry N. Hutchinson	Prehistoric Man and Beast (1896, plate III)	Moser 1998: 142	Bone-tipped harpoon
1905	Pithecanthropus	Pithecanthropus – today H. erectus (Java)	Lancelot Speed	Henry R. Knipe	Nebula to Men (1905, facing p. 165)	Moser 1998: 144	Branch/**stick**
	Early Palaeolithic Men	H. neanderthalensis	Ernest Bucknall	Henry R. Knipe	Nebula to Men (1905, facing p. 190)	Moser 1998: 144	Hand axe
	Cave Men (Hunter-Artists)	H. sapiens – Cro-Magnons	Ernest Bucknall	Henry R. Knipe	Nebula to Men (1905, facing p. 200)	Moser 1998: 145	Stone-tipped spear
1909	An Ancestor: The Man of Twenty Thousand Years Age	H. neanderthalensis (La Chapelle-aux-Saints, France)	František Kupka	Marcellin Boule	The Illustrated London News (February 1909), L'Illustration (February 1909)	Sommer 2006: 227 Moser 1998: xxiii	**Wooden or bone club (?)**
1910	*without title*	*unspecified – H. Neanderthalensis (?)*	H. Hasbach	Ludwig Wilser	Leben und Heimat des Urmenschen (1910, cover)		Branch, boulder
1911	Snowbound (Neanderthals)	H. neanderthalensis (probably inspired by La Chapelle-aux-Saints, France)	Charles R. Knight	?	*oil on canvas*	Milner 2013 King 1949: frontispeice	Stone-tipped spear
1911	Modern Man, the Mammoth-Slayer: The Briton of 170,000 Years Age	H. sapiens (Galley Hill, UK)	Amédée Forestier	Arthur Keith	The Illustrated London News (March 1911)	Sommer 2006:229 Moser 1998: 155	Stone axe, stone-tipped spear
1911	Not in the "Gorilla" Stage: The Man of 500,000 Years Ago	H. neanderthalensis (La Chapelle-aux-Saints, France)	Amédée Forestier	Arthur Keith	The Illustrated London News (May 1911)	Sommer 2006:230 Moser 1998: xxiii	Stone axe, stone-tipped spear
1912	Homo Mousteriensis	H. neanderthalensis (Le Moustier, France)	Alice B. Woodward	Henry R. Knipe	Evolution in the Past (1912, facing p. 196)		Hand axe, **stick** or bone

1915	Neanderthal man at the station of Le Moustier, overlooking the valley of the Vézère, Dordogne	H. neanderthalensis (Le Moustier, France)	Charles R. Knight	Henry F. Osborn	Men of the Old Stone Age (1915, frontispiece)	Moser 1998: xxiv	**Wooden club,** rock
1920	The Neanderthal Flint Workers	H. neanderthalensis (Le Moustier, France)	Charles R. Knight	Henry F. Osborn	*oil on canvas* in American Museum of Natural History (New York)	Osborn 1923: 11, Moser 1998: 158, Sommer 2010: 472	Hand axe, stone-tipped spear
	Cro-Magnon Artists Painting the Mammoth	H. sapiens – Cro-Magnons (Font-de-Gaume, France)	Charles R. Knight	Henry F. Osborn	*oil on canvas* in American Museum of Natural History (New York)	Osborn 1923: 14, Moser 1998: 159, Sommer 2010: 473	Wooden staff with baton de cornmandement
1921	Mousterian Cave-dwellers	H. neanderthalensis (Le Moustier, France)	Marjorie & Charles H. B. Quennell	Marjorie & Charles H. B. Quennell	Everyday life in Prehistoric Times (1921, p. 48)		Spear, hand-axe
	Poise of the Mousterian Figure	H. neanderthalensis (La Chapelle-aux-Saints, France; Neandertal valley, Germany)	Marjorie & Charles H. B. Quennell	Marjorie & Charles H. B. Quennell	Everyday life in Prehistoric Times (1921, p.51)		Stone-tipped spear
	Mousterians on the March	H. neanderthalensis (La Chapelle-aux-Saints, France; Neandertal valley, Germany)	Marjorie & Charles H. B. Quennell	Marjorie & Charles H. B. Quennell	Everyday life in Prehistoric Times (1921, p. 52)		Spear, hand-axe, **wooden club (?)**
	Types of Huts suggested by Aurignacian drawings	H. sapiens – Cro-Magnons	Marjorie & Charles H. B. Quennell	Marjorie & Charles H. B. Quennell	Everyday life in Prehistoric Times (1921, p. 70)		Stone-tipped spear
1922	Restoration by A. Forestier of the Rhodesian Man whose Skull was Discovered in 1921	H. rhodesiensis or heidelbergensis (Broken Hill, Zambia)	Amédée Forestier	J. Arthur Thompson (?)	The Outline of Science, Vol. 1 (of 4). A Plain Story Simply Told (1922, p. 176-177)		Wooden spear, rock or hand-axe
1922	The Earliest Man Tracked by a Tooth: An “Astounding Discovery” of Human Remains in Pliocene Strata	*Fictitious Hesperopithecus* (Upper Snake Creek, USA)	Amédée Forestier	Grafton E. Smith	The Illustrated London News (June 1922)		**Stick** or bone, hand-axe
1925	*?*	H. neanderthalensis (Ghar Dalam, Malta)	Amédée Forestier	Arthur Keith	The Illustrated London News (February 1925)	Weltersbach 2007: 63	**Stick**, bone, **wooden club**, rock
	Reconstructed: Australopithecus and the Rhodesian Man	H. rhodesiensis (Broken Hill, Zambia)	Amédée Forestier	Grafton E. Smith	The Illustrated London News (February 1925)	Moser 1996: 191	**Stick**
1929	Restoration of a Neanderthal Family	H. neanderthalensis (Le Moustier, France)	Frederick Blaschke	Henry Field	*diorama* in Field Museum of Natural History (Chicago)	Farrington and Field 1929: frontispiece	Hand axe, stone scraper
1933	Chellean Scene in Northern Europe about 250,000 Years Ago	*unspecified* (*Chellean period*)	Frederick Blaschke	Henry Field	*diorama* in Field Museum of Natural History (Chicago)	Field 1933: plate 1	Hand axe
	Neanderthal Family at Devil’s Tower Rock-shelter, Gibraltar	H. neanderthalensis (Devil's Tower Cave, Gibraltar)	Frederick Blaschke	Henry Field	*diorama* in Field Museum of Natural History (Chicago)	Field 1933: plate 2	**Wooden club**
1937	Urmenschen auf der Höhlenbärenjagd (Zeit des Neandertalers, letzte Zwischeneiszeit) *Prehistoric men on the cave bear hunt (time of the Neanderthals, last interglacial period)*	H. neanderthalensis	Franz Roubal	?	*school mural*		Spear, **wooden club**, hand-axe

1942	With Flint-tipped Spear, Stone Ax, and Rocks, Neanderthal Man Repel an Invader	H. neanderthalensis	Charles R. Knight	Charles R. Knight	National Geographic Magazine (February 1942)		Hand axe, stone-tipped spear, **bone club**
1950	Paranthropus and early Homo erectus	Paranthropus, H. erectus	Maurice Wilson	?	Painting in Natural History Museum (London)	Andrews & Stringer 1989: 37	**Stick**
	The Life of Homo erectus at Zhoukoudian, China	H. erectus (Zhoukoudian, China)	Maurice Wilson	?	Painting in Natural History Museum (London)	Moser 1998: 161<br>Andrews & Stringer 1989: 39	Hand axe
	Homo Neanderthalensis Hunting at Swanscombe	H. neanderthalensis (Swanscombe, UK)	Maurice Wilson	?	Painting in Natural History Museum (London)	Andrews & Stringer 1989: 43	Wooden spear
	Homo neanderthalensis, Neanderthal Man	H. neanderthalensis (Gorham's Cave, Gibraltar)	Maurice Wilson	?	Painting in Natural History Museum (London)	Andrews & Stringer 1989: 45	Wooden spear, hand axe
	Cro-Magnon Man	H. sapiens – Cro-Magnons	Maurice Wilson	?	Painting in Natural History Museum (London)	Andrews & Stringer 1989: 47	Wooden spear, spear-thrower?

References to Table S1

Andrews, Peter, and Chris Stringer. 1989. *Human Evolution: An Illustrated Guide*. Cambridge: Cambridge University Press.

Berman, Judith C. 1999. Bad Hair Days in the Paleolithic: Modern (Re)Constructions of the Cave Man. *American Anthropologist* 101 (2):288-304.

Boitard, Pierre. 1861. *Paris Avant Les Hommes, L'Homme Fossile, Etc. Histoire Naturelle Du Globe Terrestre*. Paris: Passard.

Capitan, Louis. 1903. Le peintre préhistorien Jamin. Son œuvre. *Revue de l'École d'anthropologie* XIII:311-316.

Cleuziou, Henri du. 1887. *La création de l'homme et les premiers âges de l'humanité*. Paris: C. Marpon & C. Flammarion.

Farrington, Oliver C., and Henry Field. 1929. *Neanderthal (Mousterian) Man*, *Geology Leaflet*. Chicago: Field Museum of Natural History.

Field, Henry. 1933. *Prehistoric Man: Hall of the Stone Age of the Old World*, *Anthropology Leaflet*. Chicago: Field Museum of Natural History.

Figuier, Louis. 1863. *La terre avant le déluge (1st edition)*. Paris: Libraire de L. Hachette.

———. 1866. *La terre avant le déluge (5th edition)*. Paris: Libraire de L. Hachette.

———. 1870. *L'Homme Primitif*. Paris: Libraire de L. Hachette.

Flammarion, Camille. 1886. *Monde avant la création de l'homme*. Paris: C. Marpon & C. Flammarion.

Hutchinson, Henry Neville. 1896. *Prehistoric Man and Beast*. London: Smith, Elder, and Co.

Knight, Charles R. 1949. *Prehistoric Man: The Great Adventurer*. New York: Appleton-Century-Crofts.

Knipe, Henry Robert. 1905. *Nebula to Man*. London: J. M. Dent.

Milner, Richard. 2013. Charles R. Knight: The Artist Who Saw through Time. *Fine Art Connoisseur* 10 (2).

Moser, Stephanie. 1996. Visual Representation in Archaeology: Depicting the Missing-Link in Human Origins. In *Picturing Knowledge: Historical and Philosophical Problems Concerning the Use of Art in Science*. S. B. Brian, ed. Pp. 184-214. Toronto: University of Toronto Press.

———. 1998. *Ancestral Images: The Iconography of Human Origins*. Ithaca, New York: Cornell University Press.

Osborn, Henry Fairfield. 1915. *Men of the Old Stone Age*. New York: Scribner.

———. 1923. *The Hall of the Age of Man*. New York: American Museum of Natural History.

Pettitt, Paul B., and Mark J. White. 2011. Cave men: Stone tools, Victorian science, and the 'primitive mind' of deep time. *Notes and Records of the Royal Society* 65 (1):25-42.

Quennell, Marjorie, and Charles H. B. Quennell. 1921. *Everyday Life in Prehistoric Times*. London: Batsford.

Ruddick, Nicholas. 2009. *Fire in the Stone: Prehistoric Fiction from Charles Darwin to Jean M. Auel*. Middletown, CT: Wesleyan University Press.

Rudwick, Martin J. S. 2008. *Worlds Before Adam: The Reconstruction of Geohistory in the Age of Reform*. Chicago and London: The University of Chicago Press.

Smith, Worthington G. 1894. *Man, the Primeval Savage*. London: Edward Stanford.

Sommer, Marianne. 2006. Mirror, Mirror on the Wall: Neanderthal as Image and 'Distortion' in Early 20th-Century French Science and Press. *Social Studies of Science* 36 (2):207-240.

———. 2010. Seriality in the Making: The Osborn-Knight Restorations of Evolutionary History. *History of Science* 48 (3-4):461-482.

Thomson, J. Arthur. 1922. *The Outline of Science, Vol. 1 (of 4) - A Plain Story Simply Told*. London: Waverley.

Unger, Franz. 1847. *Die Urwelt in ihren verschiedenen Bildungsperioden: XIV landschaftliche Darstellungen*. Munich: S. Minsinger and A. Geisberger.

Weltersbach, Konstanze. 2007. Homo neanderthalensis und Urmensch: Rekonstruktionen und Lebensbilder. In *Physische Anthropologie – Biologie des Menschen*. M. Kaasch, J. Kaasch and N. A. Rupke, eds. Pp. 55-69. Berlin: VWB-Verlag.

Table S2 The use of close-range clubs/sticks according to activity and frequency of use. All analyzed societies (n = 57).

Contact club usage	Violence C / B	Land animal hunting C / B	Bird hunting C / B	Marine hunting C	Fishing C
Primary	16 / 3	- / 1	2 / -	2	-
Primary?	10 / 1	2 / -	1 / 1	3	1
Secondary	15 / 1	22 / 2	9 / 1	9	21
? (uncertain)	1 / -	1 / -	1 / -	-	-
Only non-wooden or composite clubs	*3 / -*	-	-	-	*1*
No evidence	7	29	42	43	34

Note. C = clubs/sticks used only as contact weapons at close-range. B = both contact clubs and throwing sticks are used for the activity and/or the same clubs/sticks used both as contact and projectile weapons.

Table S3 The use of throwing sticks/clubs according to activity and frequency of use. All analyzed societies (n = 57).

Throwing stick usage	Violence P / B	Land animal hunting P / B	Bird hunting P / B
Primary	- / 3	- / 1	1 / -
Primary?	- / 1	1 / -	2 / 2
Secondary	- / 1	1 / 2	2 / -
? (uncertain)	- / 5	-	-
Only non-wooden or composite	- / 2	-	-
No evidence	45	52	50

Note. P = clubs/sticks used only as projectile weapons at long-range. B = both contact clubs and throwing sticks are used for the activity or the same clubs/sticks used both as contact and projectile weapons.

Table S4 The use of close-range clubs/sticks according to activity and frequency of use. Only hunter-gatherer societies (n = 39).

Contact club usage	Violence C / B	Land animal hunting C / B	Bird hunting C /B	Marine hunting C	Fishing C
Primary	11 / 1	- / 1	2 / -	2	-
Primary?	7 / 1	2 / -	1 / 1	3	1
Secondary	11 / 1	16 /2	6 / 1	7	16
? (uncertain)	-	1 / -	1 / -	-	-
Only non-wooden or composite clubs	*2 / -*	-	-	-	-
No evidence	5	17	27	27	22

Note. C = clubs/sticks used only as contact weapons at close-range. B = both contact clubs and throwing sticks are used for the activity and/or the same clubs/sticks used both as contact and projectile weapons.

Table S5 The use of close-range clubs/sticks according to activity and frequency of use. Only primarily hunter-gatherer societies (n = 18).

Contact club usage	Violence C / B	Land animal hunting C / B	Bird hunting C /B	Marine hunting C	Fishing C
Primary	5 / 2	-	-	-	-
Primary?	3 / -	-	-	-	-
Secondary	4 / -	6 / -	3 / -	2	5
? (uncertain)	1 / -	-	-	-	-
Only non-wooden or composite clubs	*1 / -*	-	-	-	*1*
No evidence	2	12	15	16	12

Note. C = clubs/sticks used only as contact weapons at close-range. B = both contact clubs and throwing sticks are used for the activity and/or the same clubs/sticks used both as contact and projectile weapons.

Table S6 The use of throwing sticks/clubs according to activity and frequency of use. Only hunter-gatherer societies (n = 39).

Throwing stick usage	Violence P / B	Land animal hunting P / B	Bird hunting P / B
Primary	- / 2	- / 1	1 / -
Primary?	-	1 / -	1 / 2
Secondary	- / 1	1 / 2	1 / -
? (uncertain)	- / 3	-	-
Only non-wooden or composite	-	-	-
No evidence	33	34	34

Note. P = clubs/sticks used only as projectile weapons at long-range. B = both contact clubs and throwing sticks are used for the activity or the same clubs/sticks used both as contact and projectile weapons.

Table S7 The use of throwing sticks/clubs according to activity and frequency of use. Only primarily hunter-gatherer societies (n = 18).

Throwing stick usage	Violence P / B	Land animal hunting P / B	Bird hunting P / B
Primary	- / 1	-	-
Primary?	- / 1	-	1 / -
Secondary	-	-	1 / -
? (uncertain)	- / 2	-	-
Only non-wooden or composite	- / 2	-	-
No evidence	12	18	16

Note. P = clubs/sticks used only as projectile weapons at long-range. B = both contact clubs and throwing sticks are used for the activity or the same clubs/sticks used both as contact and projectile weapons.

Table S8 The use of close-range clubs (C) and throwing sticks (P) according to type of hunting and whether they were used for hunting/catching animals or only for finishing animals already caught. All analyzed societies (n = 57).

**Contact club and throwing stick usage**	**Land animal hunting C / P**	**Bird hunting C / P**	**Marine hunting C**	**Fishing C**
Hunting (or HF)	21 / 5	9 / 7	9	7
? (uncertain)	5 / -	1 / -	2	1
Only finishing	2 / -	5 / -	3	14
Only on-wooden or composite	-	-	-	*1*
No evidence	29 / 52	42 / 50	43	34

Note. HF = hunting and finishing.

Table S9 Hunting techniques and delivery methods associated with clubs/sticks as recorded for each society. Divided by type of hunting.

Society	Land animal hunting	Bird hunting	Marine Hunting
San	Approach (clubbing)<br>Pursuit (clubbing)<br>Encounter (clubbing, throwing)	Approach (clubbing, throwing)<br>Encounter (throwing)	
Hadza	? (clubbing)		
Tiwi	Ambush (throwing)<br>Approach (throwing)<br>Pursuit (clubbing)<br>Encounter (clubbing)	Ambush (throwing)	
Aranda	? (throwing)	? (throwing)	
Mbau Fijians		? (throwing)	
Ainu	Disadvantage (clubbing)		? (clubbing)
Nivkh	? (clubbing)	? (clubbing)	Disadvantage (clubbing)<br>Approach (clubbing)
Yukaghir	Pursuit (clubbing)		
Ingalik	Ambush/Advantage (clubbing)<br>Pursuit (clubbing)		
Aleut			Approach (clubbing)<br>Disadvantage/Pursuit (clubbing)
Innu	Disadvantage (clubbing)<br>Encounter (clubbing)		Approach (clubbing)<br>Ambush? (clubbing)
Mi'kmaq	Ambush (clubbing)	Disadvantage (clubbing)	Approach (clubbing)
Ojibwa	Disadvantage (clubbing)	Ambush? (clubbing)<br>Pursuit (clubbing)	
Slavey	? (clubbing)		
Kaska	Ambush (clubbing)		
Eyak		Disadvantage (clubbing)	? (clubbing)
Nuxalk	Encounter? (clubbing)		Approach (clubbing)
Southern Coast Salish	Disadvantage (clubbing)		Approach (clubbing)
Yurok	Encounter (throwing)	Encounter (throwing)	? (clubbing)
Pomo	Pursuit (clubbing)<br>Encounter? (clubbing)		Approach/Pursuit (clubbing)
Yokuts	Pursuit/Disadvantage (throwing, clubbing)		
Northern Paiute	Disadvantage? (clubbing)	Disadvantage (clubbing)<br>Approach (clubbing)	
Eastern Apache	Disadvantage (clubbing)	Encounter (clubbing)	
Warao	Disadvantage (clubbing)<br>Pursuit (clubbing)		
Sirionó	Ambush (clubbing)<br>Pursuit (clubbing)<br>Encounter (clubbing)<br>Approach? (clubbing)		
Nambicuara	Encounter (clubbing)		
Canela	Disadvantage (clubbing)<br>Pursuit (clubbing)<br>Encounter (clubbing)<br>Approach (clubbing)		
Tupinamba	Disadvantage (clubbing)<br>Encounter? (clubbing)		
Xavante	Disadvantage (clubbing)	Encounter (throwing)	
Xokleng	Disadvantage (clubbing)<br>Ambush (clubbing)		
Enxet and Enlhet		Disadvantage (clubbing)<br>Approach (clubbing)<br>Ambush (throwing)	
Yahgan		Disadvantage (clubbing)<br>Approach (clubbing)<br>Pursuit (clubbing)	Approach (clubbing)

Fig. S1 Close-range clubs (red triangles) and/or throwing sticks (large circles) used in violence. Societies with no or ambiguous evidence marked with small circles.

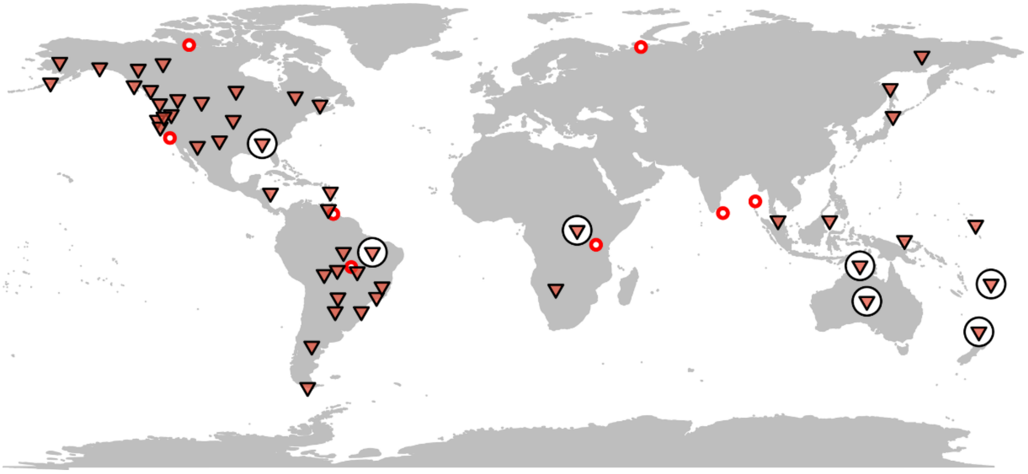

Fig. S2 Close-range clubs (orange triangles) and/or throwing sticks (green squares) used in land animal hunting. Societies with no or ambiguous evidence marked with red circles.

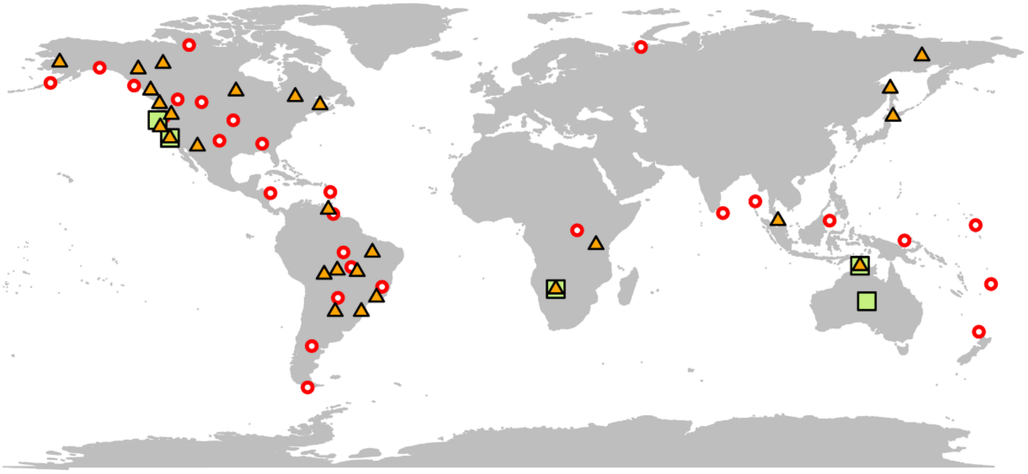

Fig. S3 Close-range clubs (orange triangles) and/or throwing sticks (green squares) used in bird hunting. Societies with no or ambiguous evidence marked with red circles.

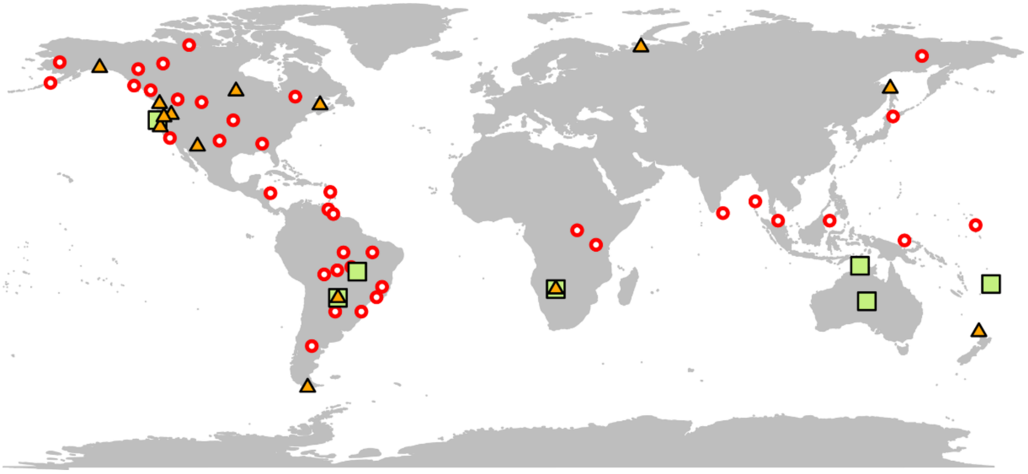

Fig. S4 Close-range clubs (orange triangles) used in marine hunting. Societies with no or ambiguous evidence marked with red circles.

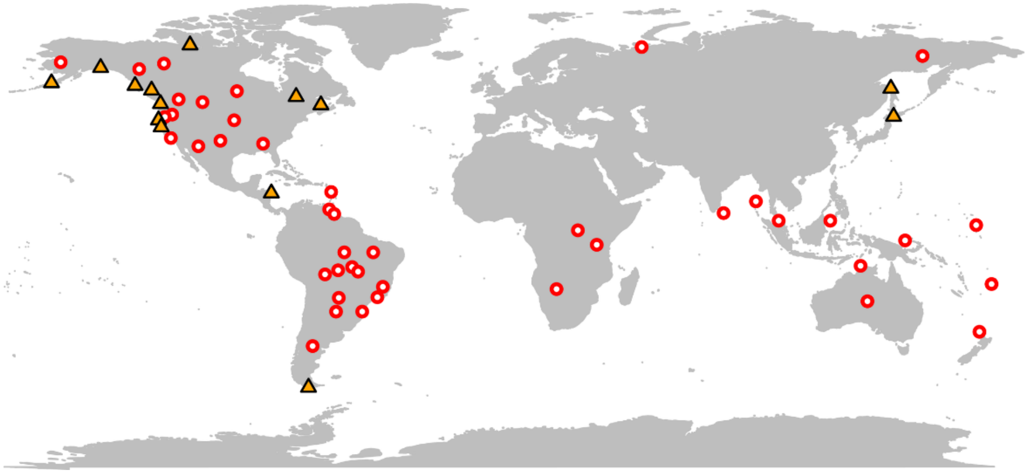

Fig. S5 Close-range clubs (orange triangles) used in fishing. Societies with no or ambiguous evidence marked with red circles.

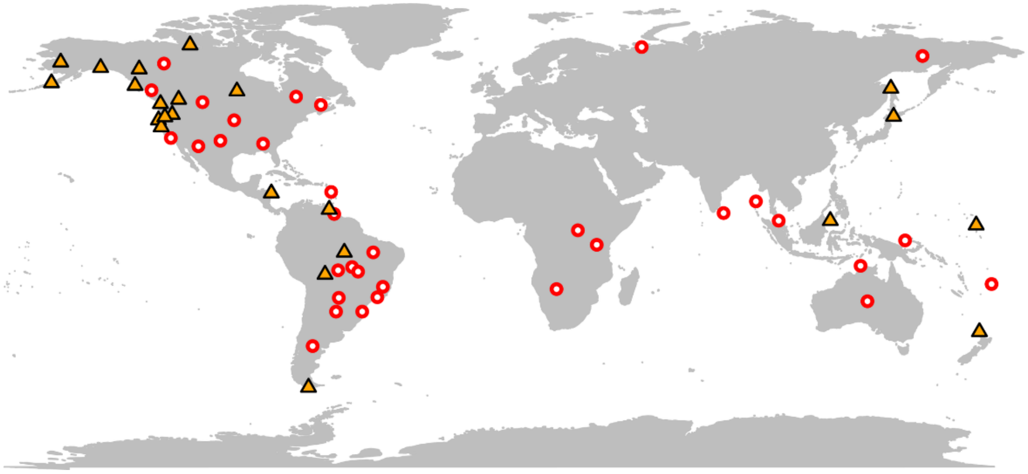

Supplement: Supplementary file 1 — Supplementary file1 (PDF 1235 KB) [file 12110_2023_9445_MOESM1_ESM.pdf]
